# Tube Insertion of Ahmed Glaucoma Valve Using a Micro-incision Scleral Tunnel Technique

**DOI:** 10.7759/cureus.75899

**Published:** 2024-12-17

**Authors:** Masaki Tanito, Hinako Ohtani, Chisako Ida, Kana Murakami, Mizuki Iida, Keigo Takagi, Akiko Harano, Kazunobu Sugihara, Sachiko Kaidzu

**Affiliations:** 1 Department of Ophthalmology, Shimane University Faculty of Medicine, Izumo, JPN

**Keywords:** ahmed glaucoma valve, allograft-free surgery, glaucoma surgery, intraocular pressure, long-tube shunt, surgical technique

## Abstract

We report three cases demonstrating the efficacy and versatility of the micro-incision scleral tunnel (MIST) technique, a novel method for Ahmed glaucoma valve (AGV) tube insertion. MIST is characterized by its small incision, sutureless approach, anterior-to-posterior tunnel creation, and allograft-free design. The technique involves creating a scleral tunnel using a 1-mm crescent knife (Bleb Knife II), allowing for secure tube placement into the anterior chamber, ciliary sulcus, or vitreous cavity. Case 1 involved a male patient in his 70s with primary angle-closure glaucoma, where the tube was inserted into the vitreous cavity. Pars plana vitrectomy was combined, achieving an intraocular pressure (IOP) of 6 mmHg without medication at three months postoperatively. Case 2 described a male patient in his 70s with secondary angle-closure glaucoma due to iridocyclitis, where the tube was inserted into the ciliary sulcus. Postoperatively, the IOP was reduced to 7 mmHg without medication at three months. Case 3 was a male patient in his teens with Axenfeld-Rieger syndrome-associated glaucoma, who underwent anterior chamber tube insertion, achieving an IOP of 8 mmHg with two medications at eight months postoperatively. In addition, postoperative anterior segment findings for the other two cases were presented. These cases demonstrate that MIST simplifies surgical procedures, eliminates the need for suturing, and achieves effective IOP control. The flexibility and promising outcomes of MIST suggest its potential as an alternative technique for tube insertion in allograft-free glaucoma tube shunt surgeries.

## Introduction

Filtration surgeries using glaucoma drainage devices with long tubes, such as the Ahmed glaucoma valve (AGV) (New World Medical, Rancho Cucamonga, CA, USA), are widely performed as intraocular pressure (IOP)-lowering procedures for glaucoma. Tube insertion sites include the anterior chamber, ciliary sulcus, and vitreous cavity, and the choice is determined based on factors such as corneal endothelial cell density, whether the eye is phakic or pseudophakic, and prior vitreous surgery [1]. To prevent conjunctival exposure of the tube, patch graft materials are used for tube coverage. Allogeneic materials such as preserved sclera, cornea, pericardium, and amniotic membrane have been employed [2,3]. To address the challenges of acquiring patch graft materials and to avoid the risks of unknown infections, autologous scleral grafting has also been practiced. Techniques include creating a scleral flap or a scleral tunnel for tube coverage [4-6]. We have previously performed surgeries by creating a half-thickness scleral flap to cover the tube, followed by suturing [7-9]. However, as part of an updated surgical approach, we introduced a new scleral coverage technique approximately one year ago. This method involves creating a smaller scleral tunnel that does not require suturing. This paper presents our novel tube insertion method, the micro-incision scleral tunnel (MIST) technique, and details its surgical procedure.

## Case presentation

Instruments required for the MIST technique

**Figure 1:**
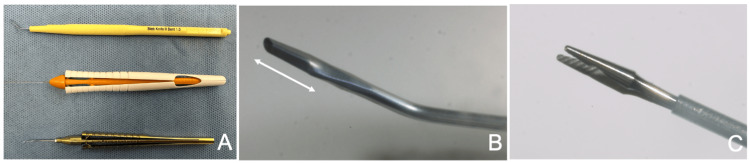
Instruments required for the micro-incision scleral tunnel (MIST) technique A: From top to bottom: Bleb Knife II (Kai Industries Co., Ltd., Gifu, Japan), Shah Xtra Grip 25G Forceps (Beaver-Visitec International, MA, USA), and Ikeda Micro Capsulorhexis Forceps (M.E. Technica, Tokyo, Japan). Either of the two forceps is required for the technique. B: Tip design of the Bleb Knife II. It has a crescent blade shape with a width of 1 mm. The blade extends approximately 3.5 mm from the tip (indicated by the double arrows). C: Tip design of the Shah Xtra Grip 25G Forceps. Forceps designed to grip surfaces, rather than just points, are more suitable for this procedure.

In addition to the standard instruments required for long-tube shunt surgery, the MIST technique necessitates a crescent knife with a 1-mm blade width (Bleb Knife II, Kai Industries Co., Ltd., Gifu, Japan) for creating the scleral tunnel (Fig. 1A, top; Fig. 1B). This knife features a blade extending approximately 3.5 mm from its tip (Fig. 1B, double arrows). In addition, forceps are required to guide the tube through the created scleral tunnel. At our institution, we use either 25G forceps typically utilized in vitreous surgery (Shah Xtra Grip 25G Forceps, Beaver-Visitec International, MA, USA) (Fig. 1A, middle; Fig. 1C) or capsulorhexis forceps commonly employed in cataract surgery (Ikeda Micro Capsulorhexis Forceps, M.E. Technica, Tokyo, Japan) (Fig. 1A, bottom). For optimal surgical handling, forceps with flat gripping surfaces are preferable over those with end-grip tips that grasp at a single point, as they provide a stronger hold when pulling the tube through the tunnel. The capsulorhexis forceps, with their curved shafts, facilitate manipulation within the anterior chamber. On the other hand, the finer tips of the vitreous forceps make it easier to insert the tube into the eye while maintaining a secure grasp.

Case 1: representative technique of MIST for pars plana (vitreous cavity) tube insertion

**Figure 2:**
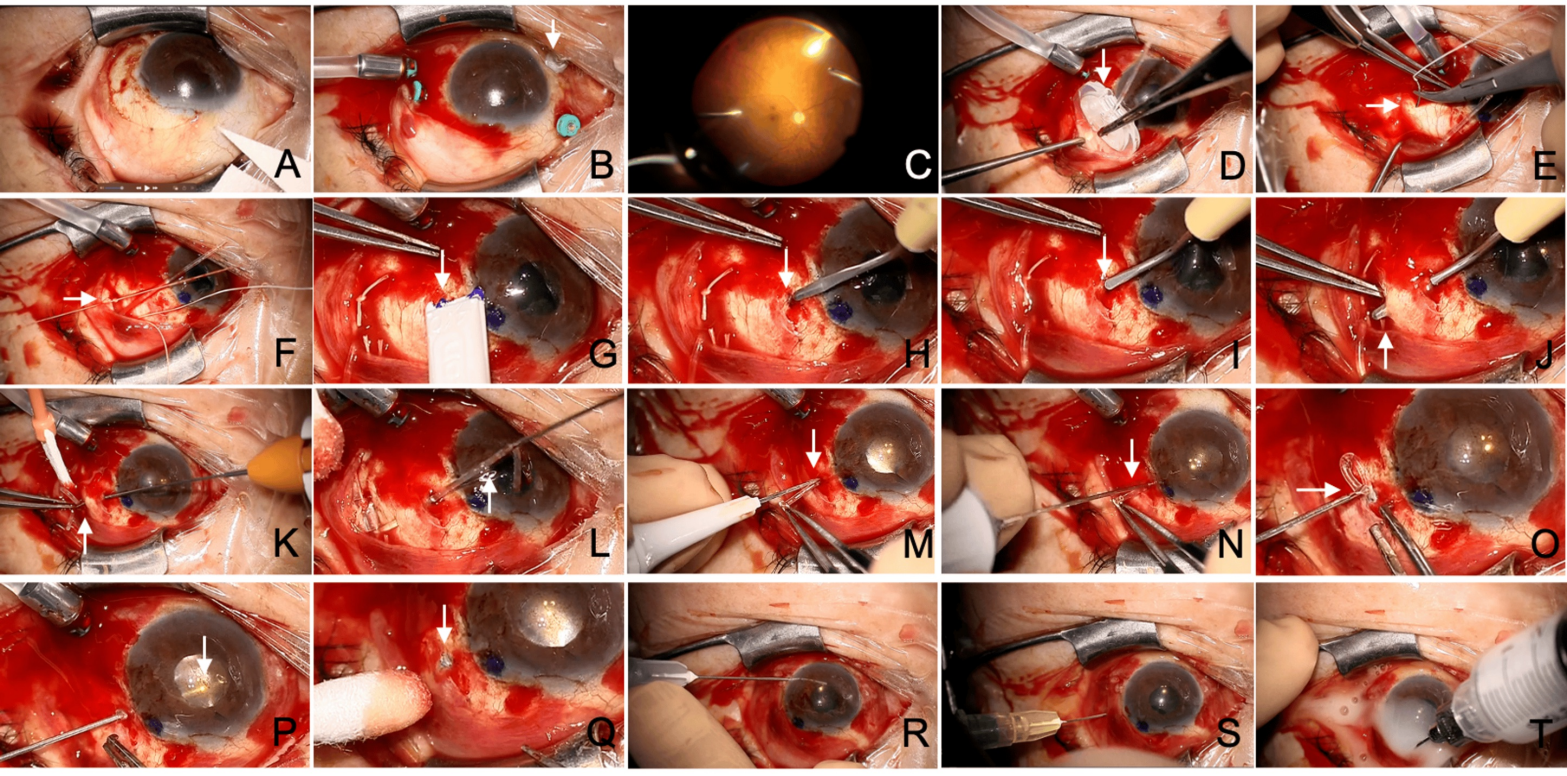
Intraoperative findings of pars plana tube insertion using the micro-incision scleral tunnel (MIST) technique (left eye) (A–T) Descriptions of each panel are provided in the main text.

A male patient in his 70s with primary angle-closure glaucoma in the left eye underwent AGV (Model FP-7) implantation due to the progression of visual field defects despite achieving an IOP of 14 mmHg with three glaucoma medications (Fig. 2, Video 1). The patient had undergone small-incision cataract surgery and Tanito microhook trabeculotomy six years prior. The vitreous was liquefied, and posterior vitreous detachment was observed. Therefore, pars plana vitrectomy was combined with AGV implantation, and the tube tip was inserted into the vitreous cavity via the pars plana. The IOP on the day after surgery was 6 mmHg. At the last observation, the IOP was 6 mmHg without glaucoma medication at three months. The corrected visual acuity was 0.9 both before and three months after the surgery.

**Video 1:** Pars plana tube insertion using the micro-incision scleral tunnel (MIST) technique (left eye)

After performing a conjunctival incision over more than one quadrant in the superotemporal area (Fig. 2A), three ports for 25G pars plana vitrectomy (blue ports) and chandelier illumination (white port, arrow) were placed (Fig. 2B). A pars plana vitrectomy was performed, clearing the peripheral vitreous with gentle indentation (Fig. 2C). After removing all vitreous ports except for the infusion port, the primed AGV was placed under the conjunctiva (Fig. 2D). A 5-0 polyester suture was passed through the sclera 8.5 mm from the limbus between the superior rectus and lateral rectus muscles (Fig. 2E, arrow). After securing two scleral sutures, the AGV plate was sutured to the sclera (Fig. 2F, arrow). A marking was made 3 mm posterior to the limbus using a surgical marker (Fig. 2G, arrow). A scleral tunnel was created using the Bleb Knife II inserted into the sclera at the marking site to a depth of half the scleral thickness (Fig. 2H, arrow). The tunnel was extended 3.5 mm, the length of the knife blade (Fig. 2I, arrow), and counterpressure was applied at the knife tip with forceps to expose the blade on the scleral surface (Fig. 2J, arrow). Using vitreous forceps passed through the tunnel, the AGV tube was grasped (Fig. 2K) and pulled through the tunnel (Fig. 2L, arrow). At the anterior end of the tunnel, perforation into the eye was made with a 25G trocar needle (Fig. 2M, arrow). The perforation was performed perpendicular to the sclera. Since the tube has a thickness of 23G, the scleral incision was slightly enlarged using the blade of the trocar needle. A 25G vitreous cutter was used to clear vitreous around the entry site (Fig. 2N, arrow). The tube, trimmed with a bevel facing posteriorly, was grasped with forceps (Fig. 2O, arrow) and inserted into the eye while maintaining the grasp (Fig. 2P, arrow). The tube’s position was confirmed to ensure it was not floating above the sclera at the insertion site (Fig. 2Q, arrow). The infusion port was removed, and the conjunctiva was sutured. To prevent postoperative hypotony, a dispersive ophthalmic viscoelastic device (OVD) (Shellgan, Santen Pharmaceutical Co., Osaka, Japan) was injected into the anterior chamber, and air was injected into the vitreous cavity. Triamcinolone acetonide was administered around the AGV plate to control inflammation and suppress postoperative scarring. Postoperatively, the patient used 1.5% levofloxacin eye drops (Viatris, Tokyo, Japan) and 0.1% betamethasone eye drops (Santen Pharmaceutical Co., Osaka, Japan) four times daily for approximately three weeks. No excessive postoperative hypotony or elevated IOP was observed. 

Case 2: representative technique of MIST for ciliary sulcus tube insertion

**Figure 3:**
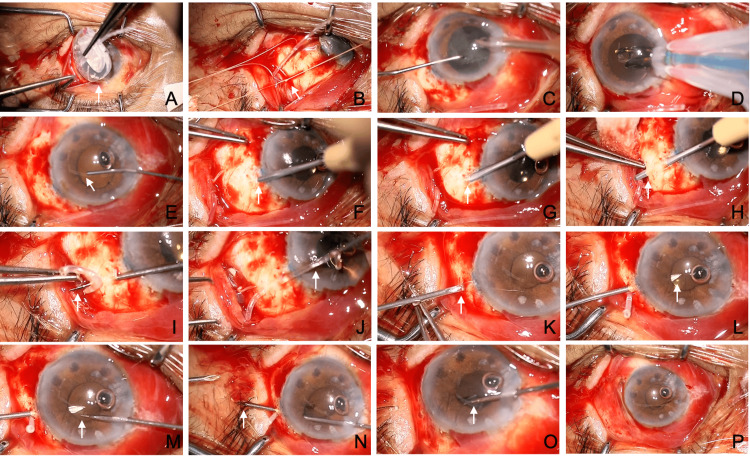
Intraoperative findings of ciliary sulcus tube insertion using the micro-incision scleral tunnel (MIST) technique (left eye) (A–P) Descriptions of each panel are provided in the main text.

A male patient in his 70s with secondary angle-closure glaucoma following iridocyclitis in the left eye underwent AGV (Model FP-7) implantation to IOP, which was 23 mmHg with three glaucoma medications (Fig. 3, Video 2). Due to peripheral retinal degeneration observed in the fundus, the tube tip was inserted into the ciliary sulcus instead of the pars plana. Simultaneous cataract surgery was performed to address the existing cataract. The IOP on the day after surgery was 5 mmHg. At the last observation, the IOP was 7 mmHg without glaucoma medication at three months. The corrected visual acuity was 0.5 before the surgery and was 0.3 at three months after the surgery.

**Video 2:** Ciliary sulcus tube insertion using the micro-incision scleral tunnel (MIST) technique (left eye)

After performing a conjunctival incision over more than one quadrant in the superotemporal area, the primed AGV was placed under the conjunctiva (Fig. 3A). Two 5-0 polyester sutures were passed through the sclera 8.5 mm from the limbus between the superior rectus and lateral rectus muscles, and the AGV plate was sutured to the sclera (Fig. 3B). A 2.2 mm corneal incision (in this case, a nasal corneal incision) was made, and small-incision cataract surgery was performed (Fig. 3C). An intraocular lens was then implanted (Fig. 3D). After removing the OVD from the anterior chamber, dispersive OVD (Shellgan, Santen Pharmaceutical) was injected into the ciliary sulcus to expand it (Fig. 3E, arrow). A scleral tunnel was created by inserting the Bleb Knife II into the sclera 1 mm posterior to the limbus to a depth of half the scleral thickness (Fig. 3F, arrow). The tunnel was extended 3.5 mm, corresponding to the length of the knife blade (Fig. 3G, arrow). Counterpressure was applied with forceps at the knife tip to expose it on the scleral surface (Fig. 3H, arrow). Using vitreous forceps passed through the tunnel, the AGV tube was grasped (Fig. 3I) and pulled through the tunnel (Fig. 3J, arrow). The tube was trimmed with a bevel facing posteriorly. A 23G sharp needle connected to dispersive OVD was inserted into the ciliary sulcus from the anterior end of the tunnel (Fig. 3K, arrow), and additional OVD was injected to expand the ciliary sulcus (Fig. 3L, arrow). From the opposite corneal incision, vitreous forceps were inserted into the anterior chamber (Fig. 3M, arrow). The tip of the vitreous forceps was guided out through the entry site created by the 23G needle (Fig. 3N, arrow). The AGV tube was grasped with the vitreous forceps, and while maintaining the grip, the forceps were retracted into the eye to draw the tube into the ciliary sulcus (Fig. 3O, arrow). The conjunctiva was then sutured. To prevent postoperative hypotony, the dispersive OVD injected into the anterior chamber was not removed. Triamcinolone acetonide was administered around the AGV plate to control inflammation and suppress postoperative scarring. Postoperatively, the patient used 1.5% levofloxacin eye drops (Viatris) and 0.1% betamethasone eye drops (Santen Pharmaceutical) four times daily for approximately three weeks. No excessive postoperative hypotony or elevated IOP was observed.

Case 3: representative technique of MIST for anterior chamber tube insertion

**Figure 4:**
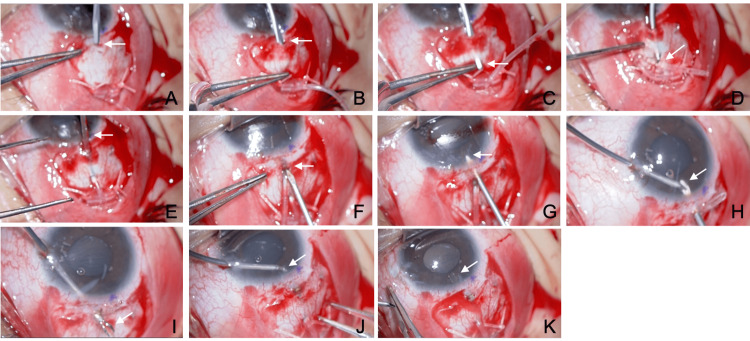
Intraoperative findings of anterior chamber tube insertion using the micro-incision scleral tunnel (MIST) technique (right eye) (A–K) Descriptions of each panel are provided in the main text.

A male patient in his teens with Axenfeld-Rieger syndrome-associated glaucoma in the right eye underwent AGV (Model FP-7) implantation due to an IOP of 31 mmHg despite using four glaucoma medications (Fig. 4, Video 3). As the patient had no cataracts and was young, the tube was inserted into the anterior chamber. A posterior embryotoxon was observed in the anterior segment, and the iris was attached at a high position in the angle; however, it was determined that there was sufficient space for tube insertion. The IOP on the day after surgery was 5 mmHg. At the last observation, the IOP was 8 mmHg with two glaucoma medications at eight months. The corrected visual acuity was 0.2 both before and 8 months after the surgery. At eight months, a slight posterior subcapsular cataract was observed.

**Video 3:** Anterior chamber tube insertion using the micro-incision scleral tunnel (MIST) technique (right eye)

After performing a conjunctival incision over one quadrant in the superotemporal area, the primed AGV was placed under the conjunctiva. Two 5-0 polyester sutures were passed through the sclera 8.5 mm from the limbus between the superior rectus and lateral rectus muscles, and the AGV plate was sutured to the sclera. A scleral tunnel was created by inserting the Bleb Knife II into the sclera 1 mm posterior to the limbus to a depth of half the scleral thickness (Fig. 4A, arrow). The tunnel was extended 3.5 mm, corresponding to the length of the knife blade (Fig. 4B, arrow). Counterpressure was applied with forceps at the knife tip to expose it on the scleral surface (Fig. 4C, arrow). Using capsulorhexis forceps passed through the tunnel, the AGV tube was grasped (Fig. 4D, arrow) and pulled through the tunnel (Fig. 4E, arrow). The tube was trimmed with a bevel facing anteriorly. A 23G sharp needle connected to dispersive OVD was inserted into the anterior chamber from the anterior end of the scleral tunnel (Fig. 4F, arrow), and additional OVD was injected to expand the anterior chamber (Fig. 4G, arrow). From a corneal side port 90 degrees away, capsulorhexis forceps were inserted into the anterior chamber and connected with the 23G needle (Fig. 4H, arrow). The 23G needle guided the forceps tip out of the eye (Fig. 4I, arrow). The AGV tube was grasped with the capsulorhexis forceps, and while maintaining the grip, the forceps were retracted into the eye to draw the tube into the anterior chamber (Fig. 4J, arrow). Proper placement of the tube within the anterior chamber was confirmed (Fig. 4K, arrow). The conjunctiva was then sutured. To prevent postoperative hypotony, the dispersive OVD injected into the anterior chamber was not removed. Triamcinolone acetonide was administered around the AGV plate to control inflammation and suppress postoperative scarring. Postoperatively, the patient used 1.5% levofloxacin eye drops (Viatris) and 0.1% betamethasone eye drops (Santen Pharmaceutical) four times daily for approximately three weeks. No excessive postoperative hypotony or elevated IOP was observed.

Representative postoperative findings

**Figure 5:**
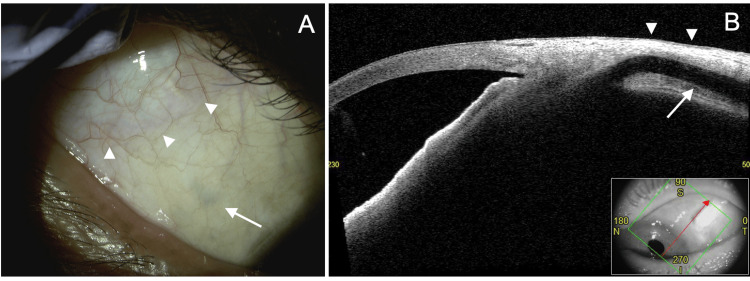
Postoperative findings after tube insertion using the micro-incision scleral tunnel (MIST) technique A: A patient in their 50s with pigmentary glaucoma. Eleven months after pars plana tube insertion in the right eye. Observation with a slit lamp reveals the tube insertion site (arrow) and the filtering bleb (arrowhead). B: A patient in their 60s with uveitic secondary glaucoma. Six months after pars plana tube insertion in the left eye. Observation with anterior segment optical coherence tomography (AS-OCT) (CASIA2 Advance, Tomey Corporation, Nagoya, Japan) shows the tube running within the sclera (arrow). No thinning is observed in the tissue overlying the tube (arrowhead).

In cases where the MIST technique was used for tube insertion, the site of tube entry into the eye can often be observed through the conjunctiva (Fig. 5A, arrow). However, the tube, including the entry point, is typically covered by adequately thick scleral, Tenon’s capsule, and conjunctival tissues (Fig. 5B, arrowhead).

## Discussion

Several methods utilizing scleral tunnels have been reported, including the use of a 24G catheter as a guide combined with a scleral patch graft [10], creating a scleral pocket with a 2.3 mm crescent knife commonly used in cataract surgery [5], and employing a diamond crescent knife for forming a scleral tunnel [6]. These techniques typically construct scleral tunnels starting from the fornix side, whereas our method involves creating the scleral tunnel from the limbus toward the fornix. This anterior-to-posterior approach offers improved maneuverability as it avoids excessive ocular rotation during the procedure. One potential risk when creating a scleral tunnel is perforation of the eye, particularly at the start of tunnel creation. However, with an anterior approach, the site of perforation, if it occurs, would likely be at the pars plana, which poses a lower risk of retinal tear formation compared to a posterior approach. Using a 1-mm crescent knife to create a small scleral tunnel is advantageous for securely fixing the tube in place. While detailed data on IOP reduction have not yet been analyzed, the IOP-lowering effect following the MIST technique appears comparable to our traditional method involving a scleral flap. In addition, as MIST does not require suturing, it simplifies the surgical procedure and contributes to reduced operative time. This aspect highlights the potential of MIST as a streamlined and effective technique for tube insertion in glaucoma drainage surgeries. In the presented cases, the tunnel creation was initiated 1 mm from the limbus for both anterior chamber and ciliary sulcus insertions. By adjusting the needle insertion angle during subsequent penetration into the anterior chamber or ciliary sulcus, the direction of tube insertion can be optimized. Therefore, it is sufficient to begin tunnel creation at “approximately” 1 mm from the limbus without requiring precise measurement.

The incidence of patch graft thinning and tube exposure following tube shunt surgery is reported to be 2-7% [6,11]. Since introducing the MIST technique in December 2023, we have performed approximately 200 cases within a year, with 90% involving vitreous cavity tube insertion. Although the postoperative follow-up period remains relatively short, we have not encountered any cases of tube exposure to date. Reported risk factors for scleral patch graft melting and tube exposure include inferior quadrant tube placement, ocular surface disease, young age, and ocular inflammatory diseases [6,11-14]. Surgeries have been performed on cases involving these risk factors as well. Therefore, we believe that MIST does not inherently increase the risk of tube exposure. However, it is known that tube exposure tends to occur more frequently beyond one year postoperatively [6,13,14]. We plan to carefully monitor postoperative outcomes following MIST to assess its long-term safety and effectiveness.

## Conclusions

We reported a novel AGV tube insertion method, named the MIST technique, characterized by its small incision, sutureless design, and anterior approach. The MIST technique is applicable for tube insertion into the anterior chamber, ciliary sulcus, or vitreous cavity. Its adoption is expected to simplify the surgical procedure and reduce operative time.
